# Environment and Health in China: Challenges and Opportunities

**DOI:** 10.1289/ehp.0901615

**Published:** 2009-12

**Authors:** Haidong Kan

**Affiliations:** Fudan Unviersity, Shanghai, China, E-mail: hdkan@shmu.edu.cn

As the largest developing country in the world, China has achieved rapid economic development, averaging an annual gross domestic product (GDP) growth rate of 10% over the past two decades. But this success comes at the cost of deterioration of the environment. China’s environmental problems, including outdoor and indoor air pollution, water shortages and pollution, desertification, and soil pollution, have become more pronounced and are subjecting Chinese residents to significant health risks.

In Chinese cities, outdoor air pollution is the biggest environmental challenge for public health. The source of air pollution in Chinese cities has gradually changed from conventional coal combustion to a mixture of coal-combustion and motor-vehicle emissions. Generally, China’s current air pollution situation is similar to to that of developed countries in the 1960s. The annual average concentrations of inhalable particles [particles < 10 μm in aerodynamic diameter (PM_10_)], sulfur dioxide (SO_2_), and nitrogen dioxides (NO_2_), the three criteria pollutants in China, were 89 μg/m^3^, 48 μg/m^3^, and 34 μg/m^3^, respectively, in 113 medium to large Chinese cities (Ministry of Environmental Protection of China 2009). Many studies have documented the adverse health effects of outdoor air pollution in China, including increases in respiratory symptoms, hospitalization, and premature mortality (Chen et al. 2004). The World Health Organization (WHO) estimated that outdoor air pollution was associated with approximately 300,000 premature deaths per year in China (Cohen et al. 2005), and Chinese scientists have given similar estimates (Zhang et al. 2008).

In rural areas of China, coal and biomass fuels are still widely used in stoves and produce substantial indoor air pollution. The evidence for adverse health effects of solid fuels is strong, including lung cancer, acute respiratory infection, and chronic obstructive pulmonary disease (Zhang and Smith 2007). The WHO estimated that solid fuels used in Chinese households cause approximately 420,000 premature deaths annually (Smith et al. 2004).

Water pollution is another cause for serious health concern in China, especially in rural areas. From 2000 to 2008, the quality of surface water worsened in northern China, where it improved slightly in southern China (Ministry of Environmental Protection of China 2009). In 2008, among 200 major rivers in China, water quality in 20.8% of 409 monitored sections was below grade V, the worst grade in the Chinese National Standard for Water Quality; water of this grade is virtually of no functional use, even for agricultural irrigation (Ministry of Environmental Protection of China 2009). Analysis of data from the 2003 National Health Services Survey (Ministry of Health of China 2004) indicates that two-thirds of the rural population does not have access to piped water. Exposure to contaminated drinking water has been associated with increasing rates of digestive cancers and infectious diseases such as hepatitis and cholera (Wu et al. 1999). The World Bank (2007) estimated that the health cost of cancers and diarrhea associated with water pollution reached approximately US$8 billion in 2003 in rural areas of China.

Other important environmental health problems in China include climate change, disposal and treatment of electronic waste, and heavy metal pollution in the soil. China is one of the countries most susceptible to the adverse effects of climate change. Although the Chinese government has paid great attention to climate change, there has been limited interest in the health impacts so far. Approximately 70% of the electronic waste generated worldwide is processed in China, posing substantial risk to human health and the environment (Ni and Zeng 2009). Also, pollution from heavy metals such as lead, mercury, chromium, cadmium, and arsenic has become increasingly prominent, seriously endangering the health of local citizens (He et al. 2009). The recent discovery of clusters of lead poisoning involving thousands of Chinese children has raised severe public concern (Watts 2009). Small-scale rural factories known as “township and village enterprises” play an important role in China’s growing pollution problems in the countryside.

China is striving to quadruple its GDP of 2000 by the year 2020, and consequently will face even more serious environmental challenges. Despite the environmental health problems described above, the Chinese government is beginning to focus on these issues and has embarked on the strategic transformation from economic development alone to environment and development in building an energy-saving and environment-friendly society. China’s economic progress can be the foundation of improvements in environmental health. In the early 1980s, the Chinese government invested US$1.6 billion annually, or 0.51% of China’s GDP, on environmental protection; in 2008, the number increased to US$66 billion, reaching 1.49% of China’s GDP (China National Bureau of Statistics 2009). In 2007, for the first time in recent years, China reported a reduction in national total emissions of chemical oxygen demand in water and SO_2_ in air, by 3.1% and 4.7% respectively, compared with the previous year. Also in 2007, China issued its first *National Environment and Health Action Plan (2007–2015)* (Ministry of Health of China 2007). The plan addresses the need to establish nationwide surveillance networks for environment and health, and for different government agencies and stakeholders to share information and take responsibility. According to the action plan, China will conduct national surveys to obtain accurate information on the nature and extent of environmental pollution and its health impact. China aims to form a comprehensive and efficient system for environmental health by 2015. Furthermore, the Chinese government will need to overcome policy and institutional barriers, such as lack of effective legislation, mechanisms for interdepartmental coordination, involvement of health authorities in environmental management, and adequate staffing at the local level.

## Note from the Editor

### International Program Grows

Readers will note that the masthead of this issue includes the names of four Regional Editors. As part of our International Program, we have identified outstanding scientists from around the world to help keep *EHP* informed about the most promising research on environmental health issues relevant to their geographical regions. We also hope the Regional Editors will work to encourage authors in their regions to submit their best work to the journal. Regional Editors are also asked to keep *EHP* informed of upcoming meetings and conferences occurring in their areas to allow the journal to focus international attention on specific needs and emerging themes. Finally, *EHP* has extended the opportunity for Regional Editors to express their thoughts and ideas concerning high-priority emerging needs or issues in editorials or commentaries. Ultimately, we hope to facilitate interactions and collaborations and the sharing of information critical to understanding the environmental issues we all face.

## Figures and Tables

**Figure:**
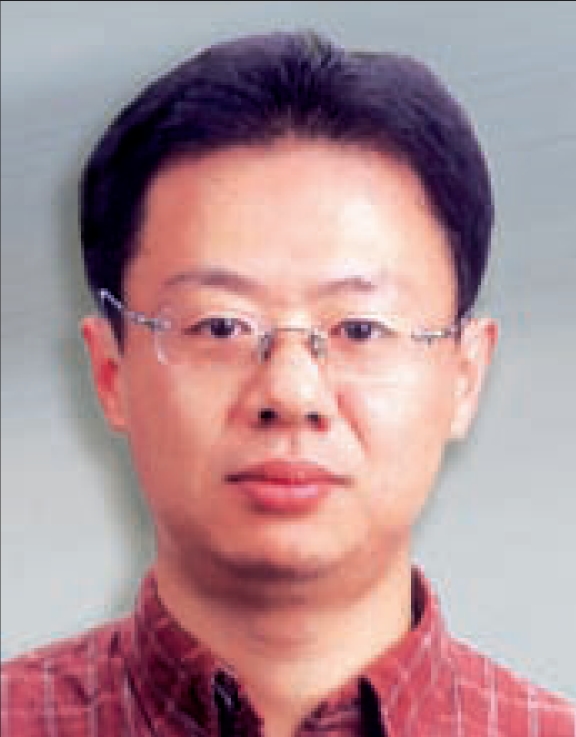
Haidong Kan
